# Adverse Reactions of First-Line Tyrosine Kinase Inhibitors in Advanced Hepatocellular Carcinoma Treatment: A Descriptive Analysis From WHO-VigiAccess

**DOI:** 10.1155/ijh/5548453

**Published:** 2025-09-12

**Authors:** Wei fan Sui, Yu xin Duan, Ze feng Cai, Yi mao Xia, Jian yun Li, Jian hua Fu

**Affiliations:** Department of Interventional Radiology, Zhenjiang First People's Hospital, Zhenjiang, Jiangsu, China

**Keywords:** adverse reaction, descriptive analysis, hepatocellular carcinoma, tyrosine kinase inhibitors, WHO-VigiAccess

## Abstract

**Aims:** To determine the safety of first-line tyrosine kinase inhibitors (TKIs) in advanced hepatocellular carcinoma (HCC) treatment by assessing the adverse drug reactions (ADRs) as reported in the World Health Organization (WHO)–VigiAccess database.

**Methods:** We compiled ADR reports for two first-line TKIs from WHO-VigiAccess with retrospective descriptive analysis, gathering data on the disease systems and symptoms associated with ADRs, as well as population and geographic characteristics of advanced HCC patients.

**Results:** A total of 63,375 ADR reports were analyzed for two first-line TKIs used in advanced HCC treatment: lenvatinib (*N* = 28,419) and sorafenib (*N* = 34,956). Gender distribution revealed male predominance for sorafenib (67.62%), whereas lenvatinib had more female reporters (53.40%). Among the 27 system organ classes, gastrointestinal disorders had the highest number of ADR reports for lenvatinib (15,683, 55.18%) and sorafenib (21,423, 61.29%). Based on gastrointestinal disorders, lenvatinib and sorafenib had similar reporting odds ratio (ROR) of 0.94 (0.96–0.92) and 0.96 (0.98–0.94) and proportional reporting ratio (PRR) of 0.95 (0.97–0.94) and 0.97 (0.98–0.95). The highest proportions of adverse reactions of diarrhea reported for the two drugs were 12% for lenvatinib and 15.74% for sorafenib.

**Conclusion:** While gastrointestinal toxicities were class-wide concerns, agent-specific risks necessitate tailored monitoring. These findings emphasized the need for vigilant monitoring and personalized management in clinical practice.

## 1. Introduction

Liver cancer, especially hepatocellular carcinoma (HCC), ranks as the sixth most prevalent cancer and the fourth leading cause of cancer-related mortality globally [1]. The advent of targeted therapies has greatly transformed the treatment of advanced HCC. Sorafenib, a multityrosine kinase inhibitor, was the initial multikinase inhibitor sanctioned for advanced HCC [2], setting a new standard of care. Later, clinical trials indicated that lenvatinib was just as effective as sorafenib regarding overall survival [3], making it another first-line treatment option for advanced HCC [4].

Sorafenib exerts dual antitumor effects. It directly curbs tumor cell proliferation by interfering with the RAF/MEK/ERK cell signaling pathway and indirectly stifles tumor growth by halting tumor neovascularization. This is achieved by blocking vascular endothelial growth factor receptor (VEGFR) and platelet-derived growth factor (PDGF) receptors [5]. Lenvatinib, on the other hand, works by inhibiting a range of kinases. These include VEGFRs 1, 2, and 3, platelet-derived growth factor receptor beta (PDGFR-*β*), fibroblast growth factor receptors (FGFR1 and 4), and fibroblast growth factor Receptor 2 (FGFR2) [6]. Although these targeted therapies offer clinical advantages, they also have unique adverse reaction profiles. Sorafenib use is linked to hand-foot skin reaction (HFSR), fatigue, and gastrointestinal issues [7]. Lenvatinib use, meanwhile, has been connected to hypertension, proteinuria, and fatigue [8].

This study sought to thoroughly analyze the adverse reaction characteristics of sorafenib and lenvatinib by utilizing data from the WHO-VigiAccess database. By comparing the adverse events of the two drugs, this study is aimed at aiding clinical decision-making and helping create personalized treatment plans for advanced HCC patients.

## 2. Methods

### 2.1. Study Design and Data Sources

This research adopted a retrospective descriptive approach to evaluate the adverse drug reactions (ADRs) of sorafenib and lenvatinib in advanced HCC patients. The evaluation leveraged data from the WHO-VigiAccess database (https://www.vigiaccess.org), which is a global database of medicinal products' potential side effects reported by national pharmacovigilance centers or drug regulatory authorities under the WHO Programme for International Drug Monitoring (PIDM). The data extracted comprised patient demographics (age and gender), regions, and ADR details like system organ classes (SOCs) and preferred terms (PTs) as per the Medical Dictionary for Regulatory Activities (MedDRA) [9].

### 2.2. Disproportionality Analysis

We utilized the reporting odds ratio (ROR) and the proportional reporting ratio (PRR) to assess disproportionate reporting [10, 11]. These tools helped evaluate issues related to TKI use and supported pharmacovigilance programs aimed at enhancing drug safety.

### 2.3. Statistical Analysis

Excel was used for descriptive statistical analysis of the ADRs linked to the two drugs. The ADR reporting rate was determined by dividing the number of reported ADR symptoms by each drug's total ADR reports. The analysis focused on common ADRs, defined as the Top 20 symptoms with the highest reporting rates. Frequencies and percentages categorized descriptive variables and highlighted similarities and differences in ADR profiles.

## 3. Results

### 3.1. General Characteristics of ADR Reports

A total of 63,375 ADR reports were analyzed for two TKIs used in advanced HCC treatment: lenvatinib (*N* = 28,419) and sorafenib (*N* = 34,956). Sorafenib, the earliest approved agent (2005), had the highest proportion of reports before 2015 (44.94%), while newer agents like lenvatinib (27.09% in 2024) showed increased reporting in recent years. Gender distribution revealed male predominance for sorafenib (67.62%), whereas lenvatinib had more female reporters (53.40%). Geographically, the Americas contributed most reports for sorafenib (35.44%), while Asia dominated reports for lenvatinib (46.38%). Additionally, 31.97% of lenvatinib reports lacked age data (Table 1).

### 3.2. SOC and Disproportionality Analysis

Among the 27 SOCs, gastrointestinal disorders had the highest number of ADR reports for lenvatinib (15,683, 55.18%) and sorafenib (21,423, 61.29%). The report rate of investigations was relatively high for lenvatinib (28.46%) and sorafenib (20.49%). General disorders and administration site conditions also had a considerable number of ADR reports for lenvatinib (10,809, 38.03%) and sorafenib (15,678, 44.85%). The report rate of hepatobiliary disorders was 5.35% for lenvatinib and 6.61% for sorafenib. In terms of respiratory, thoracic, and mediastinal disorders, lenvatinib had a report rate of 11.97% and sorafenib 45.83% (Table 2).

Based on gastrointestinal disorders, lenvatinib's ROR was 0.94 (95% CI: 0.96–0.92), and PRR was 0.95 (95% CI: 0.97–0.94). Sorafenib's ROR was 0.96 (95% CI: 0.98–0.94), and PRR was 0.97 (95% CI: 0.98–0.95) (Table 3).

### 3.3. Top 20 ADRs and Serious Adverse Events

For lenvatinib, the top ADRs included diarrhea (12.00%), fatigue (10.35%), decreased appetite (8.21%), off-label use (6.27%), and hypertension (5.65%). Sorafenib's top ADRs included diarrhea (15.74%), fatigue (7.83%), decreased appetite (7.20%), hypertension (6.06%), and weight decreased (5.42%) (Table 4).

The proportions of serious adverse reactions of death reported for these drugs were 2.39% for lenvatinib and 5.41% for sorafenib (Table 4).

## 4. Discussion

This study conducted a comprehensive descriptive analysis of the ADRs of first-line TKIs including sorafenib and lenvatinib in the treatment of advanced HCC using the WHO-VigiAccess database. The study found that there were differences in the ADR reports of different TKIs in terms of gender and geographical regions. The ADR reports of sorafenib had a higher proportion of males, while those of lenvatinib had a higher proportion of females. This gender difference might be related to the physiological characteristics of different genders [12], the rate of drug metabolism, and the varying sensitivity to the drugs. In terms of geographical regions, the Americas had a higher proportion of ADR reports for sorafenib, while Asia had a higher proportion for lenvatinib and sorafenib. This could be associated with factors such as the distribution of medical resources, the usage of the drugs [13], and the genetic background of patients in different regions [14]. Populations in different regions had different genetic polymorphisms, which could affect drug metabolism and the occurrence of ADRs [15]. Future studies could integrate real-world databases with racial stratification data to conduct a comprehensive evaluation of genetic polymorphisms' impact on TKI-related ADRs.

The results showed that the ADR profiles of these TKIs were different, yet there were some common ADRs shared among them. Gastrointestinal disorders were the most frequently reported among SOCs for two drugs, with diarrhea in the Top 20 ADRs for them, which were consistent with previous studies [7, 8]. This might be related to the fact that while TKIs exert their antitumor effects by inhibiting multiple receptor tyrosine kinases, they also impact normal cells. The proliferation and metabolism of gastrointestinal cells were disrupted [16], thereby triggering relevant ADRs. Additionally, fatigue was another common ADR, which could be associated with the interference of the drugs with cellular energy metabolism pathways, consequently affecting patients' physical strength and quality of life. Understanding the characteristics of these ADRs is of great significance for clinicians to intervene and manage them in advance during the treatment process, which can help alleviate patients' discomfort and enhance the tolerability of the treatment [17].

Through the WHO-VigiAccess database, this study also focused on serious adverse events related to TKIs, including life-threatening events, disabilities, and congenital malformations. The proportions of mortality-related ADR reports for cabozantinib, lenvatinib, regorafenib, and sorafenib were 6.36%, 2.39%, 5.96%, and 5.41%, respectively. This indicated that although TKI treatment had certain efficacy in advanced HCC, it was also associated with a relatively high risk of mortality. TKIs might affect multiple biological processes, leading to a variety of serious AEs [18, 19]. Although we reported the proportion of death-related ADRs, the lack of time-series data and cause-of-death attribution in the database made it impossible to determine which ADRs had a direct causal relationship with mortality. Future studies are recommended to incorporate clinical follow-up data and employ time-series or survival analysis methods to evaluate the impact of specific ADRs on overall survival.

In clinical decision-making, doctors should select a TKI with relatively fewer or more manageable ADRs based on individual patient conditions, such as comorbidities and drug tolerance. For instance, lenvatinib may need to be used cautiously in patients with cardiovascular disease risks [18, 20]. Additionally, for patients with severe ADRs, it may be necessary to consider dose adjustment or switching to alternative treatment plans to improve the safety and effectiveness of the treatment.

Although the WHO-VigiAccess database used in this study can provide a large amount of ADR data, there were certain limitations. Firstly, the reports in the database had incomplete information. For example, some reports lacked detailed information such as patients' age and weight, which affected the comprehensive assessment of ADR risk factors. Secondly, the reported ADRs were not necessarily caused solely by TKIs; there may be other confounding factors, such as concomitant medications and disease progression. Thirdly, the disproportionality analysis using ROR and PRR does not fully capture the true incidence of adverse reactions due to the inherent limitations of these metrics.

## 5. Conclusion

This analysis underscored gastrointestinal toxicity as a class effect of first-line TKIs in advanced HCC treatment. These findings emphasized the need for vigilant monitoring and personalized management in clinical practice.

## Figures and Tables

**Table 1:** Characteristics of adverse drug reaction (ADR) reports of four tyrosine kinase inhibitors.

	**Lenvatinib**	**Sorafenib**
Number of ADR reports	28,419	34,956
Female	15,177 (53.40%)	9282 (26.63%)
Male	12,551 (44.16%)	23,571 (67.62%)
Unknown	691 (2.43%)	2103 (6.03%)
0–27 days	1 (0.01%)	13 (0.04%)
28 days to 23 months	1 (0.01%)	15 (0.04%)
2–11 years	13 (0.05%)	134 (0.38%)
12–17 years	44 (0.15%)	176 (0.50%)
18–44 years	793 (2.79%)	2078 (5.94%)
45–64 years	7381 (25.97%)	12,561 (35.93%)
65–74 years	7025 (24.72%)	8507 (24.34%)
≥ 75 years	4075 (14.34%)	4667 (13.35%)
Unknown	9086 (31.97%)	6805 (19.47%)
Africa	40 (0.14%)	122 (0.35%)
Americas	11,557 (40.67%)	12,388 (35.44%)
Asia	13,180 (46.38%)	14,012 (40.08%)
Europe	3349 (11.78%)	8229 (23.54%)
Oceania	293 (1.03%)	205 (0.59%)
Before 2015	86 (0.30%)	15,708 (44.94%)
2016	423 (1.49%)	2723 (7.79%)
2017	819 (2.88%)	4031 (11.53%)
2018	1229 (4.32%)	2374 (6.79%)
2019	2488 (8.75%)	2051 (5.87%)
2020	2169 (7.63%)	1739 (4.97%)
2021	2572 (9.05%)	1401 (4.01%)
2022	4466 (15.71%)	1496 (4.28%)
2023	6330 (22.27%)	2016 (5.77%)
2024	7698 (27.09%)	1332 (3.81%)

**Table 2:** adverse drug reaction (ADR) number and report rate of 27 system organ classes (SOCs) of two tyrosine kinase inhibitors.

**System organ classes**	**Lenvatinib (** **N** = 28,419**)**	**Sorafenib (** **N** = 34,956**)**
Blood and lymphatic system disorders	1254 (4.41%)	2475 (7.08%)
Cardiac disorders	1494 (5.26%)	1611 (4.61%)
Congenital, familial, and genetic disorders	9 (0.03%)	33 (0.09%)
Ear and labyrinth disorders	132 (0.46%)	298 (0.85%)
Endocrine disorders	2167 (7.63%)	176 (0.50%)
Eye disorders	426 (1.50%)	573 (1.64%)
Gastrointestinal disorders	15,683 (55.18%)	21,423 (61.29%)
General disorders and administration site conditions	10,809 (38.03%)	15,678 (44.85%)
Hepatobiliary disorders	1520 (5.35%)	2311 (6.61%)
Immune system disorders	247 (0.87%)	289 (0.83%)
Infections and infestations	2735 (9.62%)	3165 (9.05%)
Injury poisoning and procedural complications	2572 (9.05%)	4491 (12.85%)
Investigations	8089 (28.46%)	7161 (20.49%)
Metabolism and nutrition disorders	4907 (17.27%)	4687 (13.41%)
Musculoskeletal and connective tissue disorders	3673 (12.92%)	4952 (14.17%)
Neoplasms benign, malignant, and unspecified (including cysts and polyps)	3021 (10.63%)	5168 (14.78%)
Nervous system disorders	5658 (19.91%)	5648 (16.16%)
Pregnancy puerperium and perinatal conditions	3 (0.01%)	5 (0.01%)
Product issues	1370 (4.82%)	1936 (5.54%)
Psychiatric disorders	2258 (7.95%)	1393 (3.99%)
Renal and urinary disorders	280 (0.99%)	476 (1.36%)
Reproductive system and breast disorders	4583 (16.13%)	4958 (14.18%)
Respiratory, thoracic, and mediastinal disorders	3401 (11.97%)	16,020 (45.83%)
Skin and subcutaneous tissue disorders	119 (0.42%)	198 (0.57%)
Social circumstances	496 (1.75%)	945 (2.70%)
Surgical and medical procedures	4081 (14.36%)	2739 (7.84%)
Vascular disorders	58 (0.20%)	79 (0.23%)

**Table 3:** Disproportionality analysis based on gastrointestinal disorders.

**Drug name**	**ROR (95% CI)**	**PRR (95% CI)**
Lenvatinib	0.94 (0.96–0.92)	0.95 (0.97–0.94)
Sorafenib	0.96 (0.98–0.94)	0.97 (0.98–0.95)

**Table 4:** Top 20 adverse drug reactions (ADRs) of two tyrosine kinase inhibitors.

**Lenvatinib ( ** **N** = 28, 419** )**		**Sorafenib ( ** **N** = 34, 956** )**	
**ADR**	**Report rate**	**ADR**	**Report rate**
Diarrhea	12.00%	Diarrhea	15.74%
Fatigue	10.35%	Rash	8.44%
Hypertension	9.39%	Fatigue	7.83%
Decreased appetite	8.21%	Decreased appetite	7.20%
Malignant neoplasm progression	7.38%	Nausea	6.41%
Nausea	7.10%	Off-label use	6.27%
Blood pressure increased	6.35%	Asthenia	5.65%
Vomiting	5.64%	Death	5.40%
Asthenia	5.55%	Hepatocellular carcinoma	5.22%
Weight decreased	4.64%	Pain in extremity	4.68%
Hypothyroidism	4.56%	Abdominal pain	4.59%
Dehydration	3.98%	Alopecia	4.31%
Headache	3.89%	Vomiting	4.30%
Rash	3.06%	Pruritus	4.07%
Stomatitis	2.88%	Weight decreased	4.05%
Arthralgia	2.79%	Hypertension	3.95%
Constipation	2.57%	Blister	3.63%
Dysphonia	2.54%	Pyrexia	3.27%
Abdominal pain	2.50%	Skin exfoliation	2.87%
Death	2.38%	Pain	2.86%

## Data Availability

The authors have nothing to report.
